# The First Case of Ischemia-Free Kidney Transplantation in Humans

**DOI:** 10.3389/fmed.2019.00276

**Published:** 2019-12-11

**Authors:** Xiaoshun He, Guodong Chen, Zebin Zhu, Zhiheng Zhang, Xiaopeng Yuan, Ming Han, Qiang Zhao, Yitao Zheng, Yunhua Tang, Shanzhou Huang, Linhe Wang, Otto B. van Leeuwen, Xiaoping Wang, Chuanbao Chen, Liqiu Mo, Xingyuan Jiao, Xianchang Li, Changxi Wang, Jiefu Huang, Jun Cui, Zhiyong Guo

**Affiliations:** ^1^Organ Transplant Center, The First Affiliated Hospital, Sun Yat-sen University, Guangzhou, China; ^2^Guangdong Provincial Key Laboratory of Organ Donation and Transplant Immunology, Guangzhou, China; ^3^Guangdong Provincial International Cooperation Base of Science and Technology (Organ Transplantation), Guangzhou, China; ^4^Department of Surgery, University Medical Center Groningen, University of Groningen, Groningen, Netherlands; ^5^Department of Anesthesiology, The First Affiliated Hospital, Sun Yat-sen University, Guangzhou, China; ^6^Immunobiology and Transplant Science Center, Houston Methodist Research Institute, Houston, TX, United States; ^7^Peking Union Medical College Hospital, Beijing, China; ^8^MOE Key Laboratory of Gene Function and Regulation, State Key Laboratory of Biocontrol, School of Life Sciences, Sun Yat-sen University, Guangzhou, China

**Keywords:** kidney transplantation, ischemia-reperfusion injury, normothermic machine perfusion, ischemia-free kidney transplantation, ischemia-free organ transplantation

## Abstract

**Background:** Ischemia-reperfusion injury (IRI) has been considered an inevitable event in organ transplantation since the first successful kidney transplant was performed in 1954. To avoid IRI, we have established a novel procedure called ischemia-free organ transplantation. Here, we describe the first case of ischemia-free kidney transplantation (IFKT).

**Materials and Methods:** The kidney graft was donated by a 19-year-old brain-dead donor. The recipient was a 47-year-old man with end-stage diabetic nephropathy. The graft was procured, preserved, and implanted without cessation of blood supply using normothermic machine perfusion.

**Results:** The graft appearance, perfusion flow, and urine production suggested that the kidney was functioning well-during the whole procedure. The creatinine dropped rapidly to normal range within 3 days post-transplantation. The levels of serum renal injury markers were low post-transplantation. No rejection or vascular or infectious complications occurred. The patient had an uneventful recovery.

**Conclusion:** This paper marks the first case of IFKT in humans. This innovation may offer a unique solution to optimizing transplant outcomes in kidney transplantation.

## Introduction

Since the first successful case of kidney transplantation was performed in 1954 (1), all transplant procedures have caused cessation of blood supply to donor organs during procurement, preservation, and implantation. The subsequent restoration of blood supply after ischemia exacerbates the initial cellular damage; this is known as ischemia-reperfusion injury (IRI) (2). Not only can IRI lead to primary non-function (PNF) or delayed graft function (DGF) in the early stage, but it can also cause chronic fibrosis and allograft rejection in the long run (3).

For decades, great efforts have been made to treat IRI (4). However, limited success has been achieved because none of the research methods is able to prevent the donor organs from experiencing initial ischemic injury. It has been shown that hypothermic machine perfusion (HMP) is superior to static cold storage (SCS) in preserving donor kidneys (5). Recently, the Leicester group has translated the use of normothermic machine perfusion (NMP) from animal studies to clinical use and performed the first kidney transplantation in a male patient after 60 min of *ex vivo* NMP in 2011 (6). Since then, the same group has used NMP for assessment and resuscitation of marginal donor kidneys (7, 8). However, since they used NMP following many hours of SCS, ischemic injury of grafts and subsequent IRI was still inevitable.

We have established a novel procedure called ischemia-free liver transplantation (IFLT) during which the donor livers can be procured, preserved, and implanted without cessation of oxygenated blood supply to the grafts (9). During IFLT, IRI can be largely avoided. In this study, we report the first case of ischemia-free kidney transplantation (IFKT).

## Case Presentation

The kidney donor was a 19-year-old male patient who died of a craniocerebral trauma. The terminal serum creatinine was 54 μmol/L. The donor and recipient were co-located in the First Affiliated Hospital of Sun Yat-sen University. The left kidney was procured after ligating the left renal artery and vein and immediately cold flushed through the left kidney artery. This graft was preserved in ice-cold University of Wisconsin solution. The right kidney was subjected to IFKT. The study protocol was approved by the Ethical Committee of our hospital. Written informed consent for the publication of the case report was obtained from the patient undergoing this ischemia-free kidney transplantation.

## IFKT Procedure

**Figure 1:**
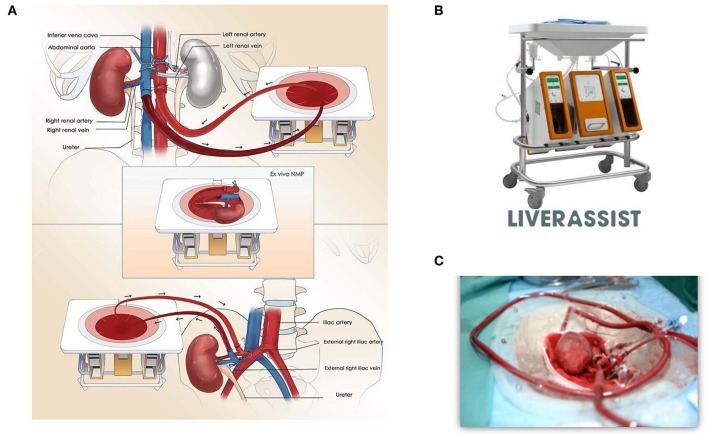
**(A)** Ischemia-free kidney transplant procedure. The diagram shows procurement, preservation, and implantation of the donor kidney without cessation of blood supply using normothermic machine perfusion. **(B)** Normothermic machine perfusion device, Liver Assist (Organ Assist, Groningen, the Netherlands). **(C)** Donor kidney circuit on the organ reservoir of the NMP device.

Figure 1A shows the technical details of the procedure. Firstly, the abdominal aorta (AA) and right renal artery, inferior vena cava (IVC), and right renal vein were well-dissected. The ureter was cut off, and a tube was placed in the ureter for urine drainage. A 34Fr caval cannula was placed in the infrarenal IVC for blood outflow to the organ reservoir of the Liver Assist device (Organ Assist, Groningen, the Netherlands, Figure 1B). A straight 20Fr cannula was inserted into the infrarenal AA of the donor. Subsequently, the arterial cannula was connected to the artery perfusion line of Liver Assist, and the suprarenal AA was blocked. The venous drainage of suprarenal IVC was also blocked. After the circuit of *in vivo* NMP was established, the right kidney was harvested and moved to the organ reservoir for *ex vivo* NMP (Figure 1C). Simultaneously, the donor liver was procured after cold flush via a cannula in the superior mesenteric vein. We sutured part of the suprarenal IVC with 6-0 Prolene and reduced its diameter to 1.5 cm on the organ reservoir of NMP *ex vivo*.

**Figure 2:**
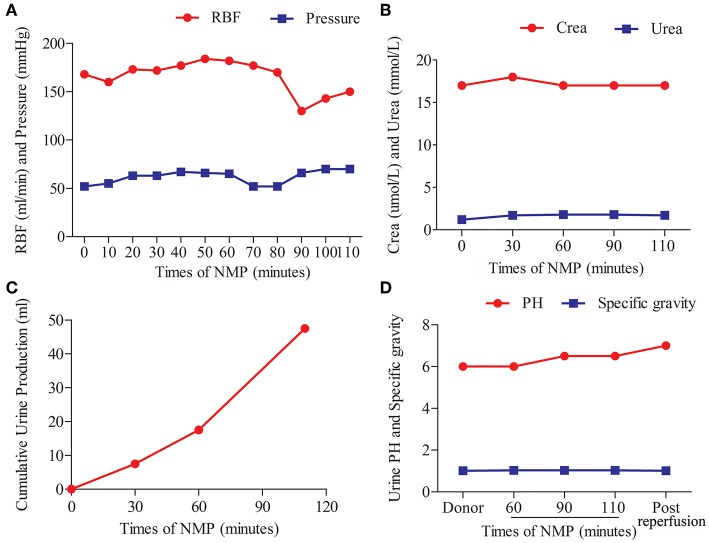
Normothermic machine perfusion and allograft viability. **(A)** Arterial flow rates and pressure. **(B)** Creatinine (Crea) and urea concentration in the perfusate. **(C)** Volume of urine production during machine perfusion. **(D)** pH values and specific gravity levels of the urine produced before procurement, during machine perfusion, and post-reperfusion.

The kidney graft underwent continuous NMP for 110 min. The perfusate components are shown in Table 1. The perfusate was warmed up to 37°C, and the oxygenator was supplied with air. The viability of the graft was assessed based on the appearance of graft and perfusion flow, as well as urine production. No creatinine was added to the perfusate for the graft function test. The perfusion pressure (52–70 mmHg) and flow (130–184 mL/min) were stable during the whole procedure (Figure 2A). The creatinine and urea levels in the perfusate were stable and low (Figure 2B). The kidney graft continued to produce urine during the whole procedure (Figure 2C). The pH values and specific gravity of the urine produced before procurement were comparable to those produced during NMP and after reperfusion (Figure 2D).

**Table 1:** Components of the perfusate solution.

**Components**	
Crossed-matched leucocyte-depleted washed red cells	1,000 mL
Sodium, potassium, magnesium, calcium, and glucose injection	600 mL
Succinylated gelatinor	400 mL
5% sodium bicarbonate	50 mL
Heparin	25,000 units
Metronidazole	0.5 g
Miropeen for injection	1 g
10% calcium gluconate	8 mL
Compound amino acid injection	40 mL
Dexamethason	15 mg
Insulin	100 units
20% mannitol	20 mL

The donor kidney was then moved from the reservoir and placed in the right iliac fossa of the recipient so that an *in vivo* NMP circuit was re-established. The donor suprarenal AA and suprarenal IVC were anastomosed to the recipient right external iliac artery and vein in an end-to-side fashion using 6-0 Prolene. During the vascular anastomoses, caution was taken to prevent twisted perfusion lines. After that, the clamp on the donor suprarenal AA was released so that recipient blood supply for the donor kidney was established. In the meantime, NMP was stopped. Around 50 mL perfusate was flushed out of the kidney, followed by release of the clamp on the donor suprarenal IVC. The cannulas in the IVC and AA were removed, and the infrarenal IVC and AA were sutured closed. Finally, after withdrawal of the draining tube, the donor ureter was anastomosed to the bladder of the recipient. The recipient operation time was 135 min.

## Outcomes

**Figure 3:**
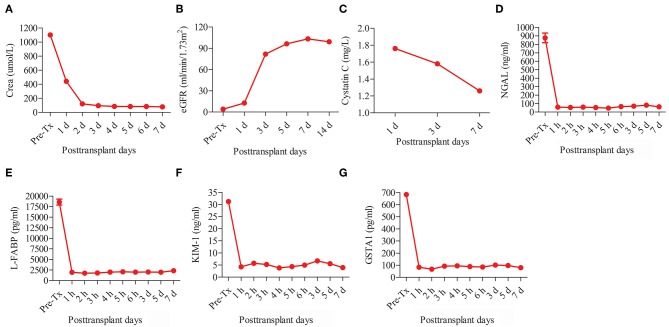
Post-transplant renal function and serum biomarker levels of kidney injury of the recipient. The renal function tests including **(A)** creatinine (Crea) and **(B)** estimated glomerular filtration rate (eGFR). The serum biomarker levels of kidney injury including **(C)** cystatin C, **(D)** neutrophil gelatinase-associated lipocalin (NGAL), **(E)** liver fatty acid-binding protein (L-FABP), **(F)** kidney injury molecule-1 (KIM-1), and **(G)** glutathione s-transferase alpha 1 (GSTA1).

The recipient was a 47-year-old man with diabetic nephropathy who had been on hemodialysis for more than 2 years. The recipient had immediate graft function, and the serum creatinine levels fell from 1100 μmol/L pre-transplantation to 95 μmol/L on post-operative day (POD) 3 (Figure 3A). The mean urine output over the first 5 days post-transplantation was 4,987 mL/day compared with a pre-transplant daily output of <100 mL.

The post-transplant estimated glomerular filtration rate (eGFR) increased from 3.99 mL/min/1.73m^2^ pre-transplantation to 103.28 mL/min/1.73m^2^ on POD 7 (Figure 3B). The post-transplant level of cystatin C (Cys C) was quite low (1.76 mg/L on POD 1 and 1.26 mg/L on POD 7) (Figure 3C). The levels of other serum kidney injury biomarkers (10–13), including neutrophil gelatinase-associated lipocalin (NGAL), liver fatty acid-binding protein (L-FABP), kidney injury molecule-1 (KIM-1), and glutathione s-transferase alpha 1 (GSTA1), all sharply decreased after IFKT (Figures 3D–G). No acute rejection, renal artery or renal vein thrombosis, or infectious complications occurred. The patient was discharged with a serum creatinine of 80 μmol/L on POD 15.

## Discussion

Kidney transplantation is inevitably associated with IRI, which can cause acute cellular injury and renal dysfunction (14–18). Several methods to ameliorate IRI have been proposed, including ischemic pre-conditioning, pharmacological interventions, protective gases, and gene and stem cell therapies (4). However, few of these methods have been translated into clinical practice. In contrast to the intention to “treat” IRI in earlier studies (4), our group focused on “preventing” IRI. We have shown that the concept of ischemia-free organ transplantation (IFOT) is feasible, safe, and effective in liver transplantation (9). Histological analysis, inflammatory cytokine production, and pathway analysis suggested that IRI is largely avoidable.

In this case report, we showed for the first time that IFOT can be expanded to kidney transplantation. The kidney was continuously functioning, with urine production during procurement, preservation, and implantation during IFKT. The serum creatinine dropped rapidly to normal range within 3 days, and eGFR increased rapidly post-transplantation. A recent study indicated that CysC can maintain its predictive power for adverse outcomes in patients with no meaningful GFR (19). The serum CysC level and the other renal injury markers were quite low post-transplantation. Since the kidney experienced ongoing NMP, for the safety of the recipient, no biopsy was obtained to confirm the absence of IRI. The recipient had an uneventful recovery without infectious or surgical complications.

Successful IFKT is established based on efficient NMP. To our knowledge, all studies concerning kidney NMP in humans are from the Leicester group (6, 7, 20, 21). They have reported that NMP can resuscitate human kidneys deemed untransplantable and can help assess kidney quality (20, 21). Moreover, the use of NMP is associated with significant decrease in DGF (6). However, the 1-h NMP is preceded by a period of cold storage after procurement and followed by a cold flush before implantation. Therefore, ischemic injury and subsequent graft IRI are still unavoidable. In contrast, IFKT maintains continuous oxygenated blood supply to the kidney, and thus IRI is probably avoided, and this may further reduce the DGF rate and improve long-term outcome after kidney transplantation, although the donor and recipient need to be at the same hospital because of the limited NMP time. This method may be more useful in expanded criteria donor (ECD) kidneys, since ECD kidneys are more susceptible to IRI. It would be highly interesting to compare the transplant outcomes of IFKT, kidney transplantation using NMP as a preservation method, and conventional kidney transplantation in the future.

Undoubtedly, there are several limitations to this study. Firstly, the donor and recipient are usually not co-located. Therefore, a portable kidney NMP device is required for this procedure. However, to our knowledge, this kind of device is not commercially available. Secondly, it is difficult to conduct IFKT of both donor kidneys for two recipients. In addition, although serum kidney injury marker levels have been tested to assess IRI severity, a biopsy of the graft should be done to confirm the absence of IRI. Finally, the kidney in this case was from a young standard brain-death donor. No significant difference in transplant outcomes and renal graft function was documented between the two recipients of the left and right renal grafts. The potential benefits should be tested in a prospective, randomized controlled study in a predefined subgroup of ECD kidneys.

In summary, we report here that IFKT is technically feasible. This innovation might be able to optimize transplant outcomes and maximize graft utilization.

## Data Availability Statement

The raw data supporting the conclusions of this manuscript will be made available by the authors, without undue reservation, to any qualified researcher.

## Ethics Statement

The studies involving human participants were reviewed and approved by The Ethical Committee of First Affliated Hospital of Sun Yat-sen University. The patients/participants provided their written informed consent to participate in this study.

## Author Contributions

XH and ZG conceived of the IFKT, they designed the procedure, performed the operation, enrolled the patient into the study, and wrote the report. GC and ZZhu designed the procedure and study protocol, followed up with the patient, analyzed the data, and critically revised the report. ZZha, XY, MH, and QZ assisted with the design of the procedure, preclinical preparation, the operation, analyzed the data, and revised the report. YZ, YT, SH, LW, OL, XW, and CC assisted with the design of the procedure, preclinical preparation, and the operation. LM, XJ, and CW assisted with the design of the procedure, the operation, preclinical preparation, and collected clinical data. XL and JH assisted with the design of the procedure, study protocol, and critically revised the manuscript. JC assisted analysis of the data and revision of the article.

### Conflict of Interest

The authors declare that the research was conducted in the absence of any commercial or financial relationships that could be construed as a potential conflict of interest.
